# Wide Excision and Flap Reconstruction in Perineal Extramammary Paget’s Disease Patients

**DOI:** 10.3390/medicina62071291

**Published:** 2026-07-03

**Authors:** Seung Yun Oh, Sodam Yi, Seokchan Eun

**Affiliations:** 1 Faculty of Medicine, Dentistry and Health Sciences, The University of Melbourne Medical School, Parkville, Melbourne, VIC 3010, Australia; seungyuno@student.unimelb.edu.au; 2 Department of Plastic and Reconstructive Surgery, Seoul National University Bundang Hospital, Seoul National University College of Medicine, 82 Gumi-ro, 173 Beon-gil, Bundang-gu, Seongnam 463-707, Republic of Korea; lsds4774@snubh.org

**Keywords:** extramammary Paget’s disease, EMPD, superficial circumflex iliac artery perforator flap, anterolateral thigh flap

## Abstract

*Background and Objectives*: Extramammary Paget’s Disease (EMPD) of the perineal region is a rare intraepidermal adenocarcinoma requiring wide excision, resulting in extensive defects that are challenging to reconstruct while preserving contour and function. This descriptive case series evaluated a reconstructive selection strategy using pedicled superficial circumflex iliac artery perforator (SCIP) flaps and pedicled anterolateral thigh (ALT) flaps for perineal defects following wide excision of EMPD. *Materials and Methods*: This retrospective case series reviewed patients with perineal EMPD who underwent wide excision followed by reconstruction using pedicled SCIP flaps or pedicled ALT flaps. Patient demographic and lesion characteristics, operative and flap characteristics, post-reconstruction complications, oncologic outcomes, and satisfaction were analyzed. *Results*: 15 patients (mean age 63 years, SD 7.3) were included in this case series. Ten patients underwent reconstruction using pedicled SCIP flaps (mean 106 cm^2^, SD 23.3), and five patients with pedicled ALT flaps (mean 245.2 cm^2^, SD 41.2). All flaps survived, but one patient developed limited partial necrosis managed with secondary healing. During a mean follow-up of 17.7 months (SD 1.3), one patient (6.7%) developed recurrence and eventually distant metastasis resulting in death. Among the 14 surviving patients, 13 (92.9%) reported overall satisfaction with cosmetic and functional outcomes assessed using a non-validated ordinal scale. *Conclusions*: Pedicled SCIP and ALT flap reconstruction provides reliable, well-vascularized tissue coverage for perineal EMPD defects and achieves generally favorable short-term outcomes. The choice between flap types should be tailored to the defect size, location, and patient characteristics.

## 1. Introduction

Extramammary Paget’s disease (EMPD) is an uncommon but low-risk intraepidermal adenocarcinoma arising from apocrine gland-bearing skin, and often involves the perianal and perineal region in both males and females [1,2,3]. Perineal EMPD typically affects elderly patients and is frequently diagnosed late, with numbers up to 3 to 4 years, due to its nonspecific clinical features, which are often mistaken for eczema, dermatitis, or tinea cruris [4,5].

Wide local excision with histologically negative margins remains the standard treatment for localized EMPD, but resection often leaves extensive superficial defects involving the penis, scrotum, perineum, and adjacent pubic skin [4,5,6,7]. Reconstruction after EMPD excision can be challenging due to the necessity to restore durable coverage, preserve elasticity for penile function and testicular thermoregulation, and maintain acceptable cosmetic contour in a psychologically sensitive area [8,9,10]. Various reconstructive options have been described for perineal defects, ranging from split-thickness skin grafts and local advancement flaps for smaller, well-approximated defects, to musculocutaneous flaps for larger or deeper defects requiring vascularized bulk, and perforator-based flaps for thin, pliable coverage with reduced donor-site morbidity [11,12,13,14]. Thin skin grafts may be acceptable in selected settings; however, larger grafted areas can be limited by contracture, contour mismatch, and erectile dysfunction, whereas myocutaneous flaps, due to their bulky nature, can increase scrotal temperature and cause contour deformity [15,16].

The choice of flap for EMPD reconstruction depends on the defect size, depth, and location, as well as the need to balance durable coverage with preservation of perineal contour and function. The anterolateral thigh (ALT) perforator flap has been a versatile option for various reconstructive purposes and has been successfully applied in perineal reconstruction as a pedicled flap [17,18]. The pedicled ALT flap can provide a reliable vascular supply and substantial tissue volume for large, deep, or contour-demanding defects, including exposed testicles or pubic involvement, but it may be relatively bulky for fine perineal contour matching in some patients [19,20,21]. The tensor fascia lata (TFL) flap offers reliable, well-vascularized coverage for large groin or perineal defects and is particularly useful when stronger tissue coverage is needed, although its bulky nature and arc of rotation can be a limitation for superficial and medial perineal defects [11,22,23,24]. Perforator-based flaps, particularly internal pudendal artery perforator (IPAP) flaps, have become important options for large perineal defects because they provide thin, pliable, well-vascularized tissue while preserving the underlying muscle and reducing donor site morbidity [14,25]. The superficial circumflex iliac artery perforator (SCIP) flap is similarly attractive for penoscrotal reconstruction, as it provides thin, pliable tissue close to the operative field, making it useful when contour preservation is a priority [9,26,27,28].

In this study, we selectively employed pedicled SCIP flaps for smaller defects requiring thin coverage and pedicled ALT flaps for larger or deeper defects requiring greater tissue volume following wide excision of perineal EMPD. This study aims to describe a reconstructive selection strategy using pedicled SCIP and ALT flaps for perineal EMPD defects, reporting on flap viability, complications, patient satisfaction, and short-term oncologic outcomes.

## 2. Materials and Methods

**Figure 1:**
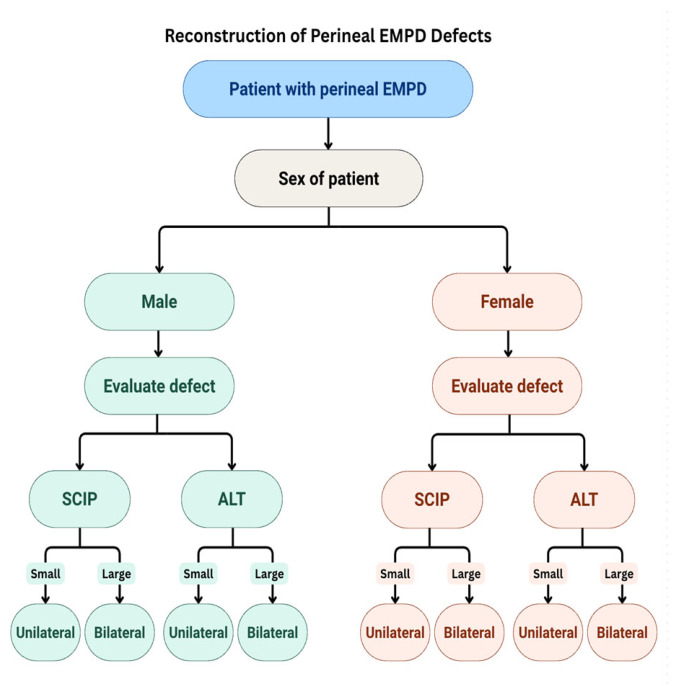
Algorithm for reconstruction of perineal EMPD defects. Superficial circumflex iliac artery perforator (SCIP); Anterolateral thigh (ALT). Flap selection is based on defect size, topography, depth, and tissue volume requirements, with unilateral reconstruction favored for smaller defects and bilateral reconstruction considered for larger or more extensive defects.

This descriptive retrospective case study included male and female patients with perineal EMPD who underwent consecutive wide local excision and pedicled flap reconstruction using either SCIP or ALT flaps between January 2023 and December 2024. All consecutive patients meeting the inclusion criteria during this period were included; no patients were excluded based on reconstructive method, as pedicled SCIP and ALT flaps were the only regional pedicled flap options used at our institution for this indication during this study’s timeframe. The inclusion criteria for this case series were: (1) histologically confirmed EMPD involving the perineal or genital region; (2) lesions involving the pubic region, penis, perineum, and/or scrotum requiring wide excision; and (3) reconstruction using a pedicled SCIP or ALT flap. Patients with distant metastasis, severe systemic disease precluding surgery, or incomplete medical records were excluded. Figure 1 entails the algorithm for perineal EMPD reconstruction.

### 2.1. Flap Selection Criteria

The choice between pedicled SCIP and pedicled ALT flap was determined by objective criteria, including defect topography (penile shaft, scrotum, perineum, or inguinal region), defect depth (superficial vs. deep/three-dimensional), tissue volume requirement, and the feasibility of primary donor-site closure. Pedicled SCIP flaps were preferred for defects requiring thin, pliable coverage of the penile shaft, scrotum, and adjacent pubic region, where tissue bulk would compromise contour or thermoregulatory function [9,27,28]. Pedicled ALT flaps were selected for infected wound or larger defects where greater tissue volume and arc of rotation were required to achieve tension-free closure, particularly for combined perineal and inguinal defects [12,19,21,29,30]. Lesion extent was documented, and defect size was estimated to guide reconstructive planning. Preoperative computed tomographic angiography was performed to map the superficial circumflex iliac artery and thigh perforators to identify suitable pedicles for flap elevation [31,32].

### 2.2. Surgical Technique

#### 2.2.1. Lesion Excision

Under general anesthesia, patients were positioned supine for anterior or lateral lesions and in lithotomy for perineal lesions [17,21,25]. The clinical margin of the lesion was outlined, extending 2–3 cm beyond the visible edge, depending on edema and suspected subclinical spread. Excision depth extended to the deep fascial layer of the penile region, the outer fascial layer of the spermatic cord in the scrotum, and the deep subcutaneous fat in the pubic and inguinal regions. After tumor removal, the wound was irrigated, and hemostasis was achieved. Frozen-section biopsies were obtained from 6 to 7 sites along the lateral margins and the base of the lesion. When margins were positive, an additional 2 cm re-excision was performed until all margins were negative.

#### 2.2.2. Pedicled SCIP Flap Design and Elevation

For SCIP flap reconstruction, the superficial (medial) branch of the superficial circumflex iliac artery (SCIA) was identified preoperatively using multidetector computed tomographic angiography and confirmed intraoperatively with a handheld Doppler device [9,28]. The superficial branch was preferentially used to avoid injury to the lateral femoral cutaneous nerve and to simplify dissection [33,34]. The flap was designed over the groin region, with dimensions approximately 2–3 cm larger than the defect, while maintaining a pedicle width of 2–3 cm. The pivot point was centered on the dominant perforator closest to the defect, and the pedicle length was confirmed to allow tension-free insetting [16,27,34]. The flap was raised in the superficial fascial plane, superficial to the deep fascia and deep to the Scarpa’s fascia. The flap was then transposed as a pedicled flap through a subcutaneous tunnel or via an open incision to reach the perineal defect, where it was inset without tension using layered closure. Suction drains were placed beneath the flap and at the donor site. The donor site was then primarily closed in all cases.

#### 2.2.3. Pedicled ALT Flap Design and Elevation

For ALT flap reconstruction, perforators arising from the descending branch of the lateral circumflex femoral artery were mapped preoperatively using computed tomographic angiography and confirmed intraoperatively with a handheld Doppler device [20,31]. The flap was designed over the anterolateral thigh, with the perforator at its center, with dimensions tailored to the defect size. An exploratory incision along the medial border of the flap was first made to identify and confirm suitable perforators [18,19]. Once confirmed, the flap was then raised in the subfascial plane and then dissected proximally along the pedicle to achieve an adequate arc of rotation for tension-free transfer to the perineal defect [21,25,29].

### 2.3. Postoperative Management and Follow-Up

Ambulation was limited for 3–5 days to minimize tension on the flap. Drains were removed when output decreased below 30 mL per day, and skin sutures were removed 10–14 days postoperatively. Patients were followed at 1 and 3 months, then every 3–6 months for at least 1 year or more if clinically indicated. Outpatient follow-up focused on flap-related complications, including total flap loss, flap necrosis, contracture, functional impairment, donor-site healing, and systemic complications. Oncologic outcomes were also evaluated based on recurrence, distant metastasis, and tumor-related death. Finally, patient-reported satisfaction was assessed based on overall cosmetic and functional outcomes.

### 2.4. Data Collection and Statistical Analysis

Collected variables for analysis included age, comorbidities prior to surgery, lesion location, defect size, flap type, flap dimensions, operative time, flap-related complications, recurrence, survival, and patient-reported satisfaction. Patient-reported satisfaction was assessed only among surviving patients at outpatient follow-up by the attending surgeon, using a non-validated four-point ordinal scale: 1 (poor), 2 (satisfactory), 3 (good), 4 (excellent). Given the limited sample size of this study, descriptive analysis was conducted to summarize the data over a formal inferential statistical comparison between the two flap groups. All analyses were performed on IBM SPSS Statistics (ver 26.0). Normal continuous variables were expressed as means with standard deviation (SD) and range, whereas non-normal continuous variables were expressed as median with interquartile ranges (IQR) and range. Categorical variables were expressed as frequencies with percentages.

## 3. Results

### 3.1. Patient Characteristics

In this case series, a total of 15 patients were included, with a mean age of 63 years (SD 7.3, range 51–78), of whom 73.3% were male. The median duration of symptoms before diagnosis was 14 months (IQR 9–24, range 5–84). One patient (6.7%) had enlarged inguinal lymph nodes, with one of the nodes showing metastatic involvement on pathological examination. Further patient and flap characteristics are summarized in Table 1. After wide excision with frozen-section margin control, defect sizes ranged from 5 × 6 cm to 16 × 18 cm. The mean defect area was 72.6 cm^2^ (SD 54, range 18–234). 10 patients underwent reconstruction using pedicled SCIP flaps (mean flap size 106 cm^2^, SD 23.3, range 30–240), and 5 patients underwent reconstruction using pedicled ALT flaps (mean flap size 245.2 cm^2^, SD 41.2, range 120–338). In the SCIP group, the superficial SCIA branch was utilized as the pedicle in all flaps. In the ALT group, perforators from the descending branch of the lateral circumflex femoral artery were used in all cases. Autologous full-thickness skin grafts were additionally used in one patient in the ALT group to cover residual defects not amenable to primary closure or flap coverage. Further operative details and flap characteristics are summarized in Table 2. Patient-level information on variables assessed can be found in the Supplementary Table S1.

### 3.2. Flap Survival and Complications

All pedicled flaps survived without total flap loss. One patient (6.7%) in the SCIP group developed limited partial necrosis at the distal flap tip, representing approximately 2% of the total flap area. This area was successfully managed with debridement and local wound care without compromising final function or aesthetic outcomes. Among patients who received combined skin grafting, one (6.7%) experienced approximately 5% skin graft loss, which healed with conservative management. There were no cases of significant donor-site morbidity or major complications such as severe scar contracture or voiding dysfunction. No patients developed deep vein thrombosis or pulmonary embolism. Donor sites in the SCIP group were primarily closed in all cases. In the ALT group, split-thickness skin grafts were required for donor-site closure in one patient due to wider flap harvest. Further postoperative characteristics and outcomes are summarized in Table 3.

### 3.3. Oncologic Outcomes and Patient Satisfaction

**Figure 2:**
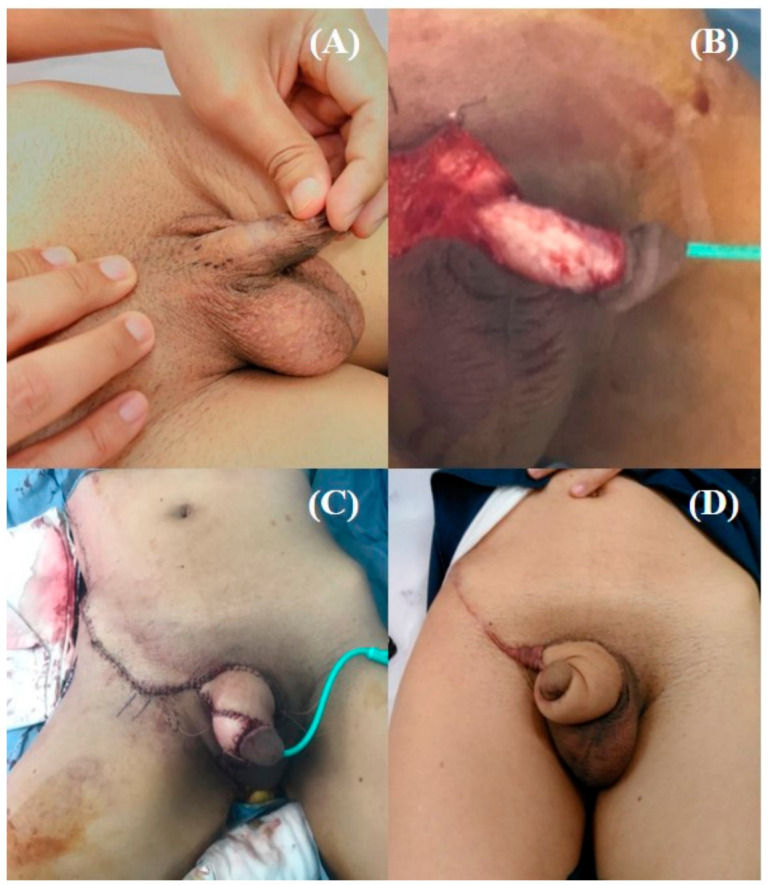
(**A**) Unilateral pedicled SCIP case of a 51-year-old male with punch biopsy-proven EMPD at the penile shaft. (**B**) Wide excision of the penile shaft skin, including the Dartos fascia. (**C**) Unilateral pedicled SCIP flap from the right inguinal area. (**D**) 3-month postoperative view.

**Figure 3:**
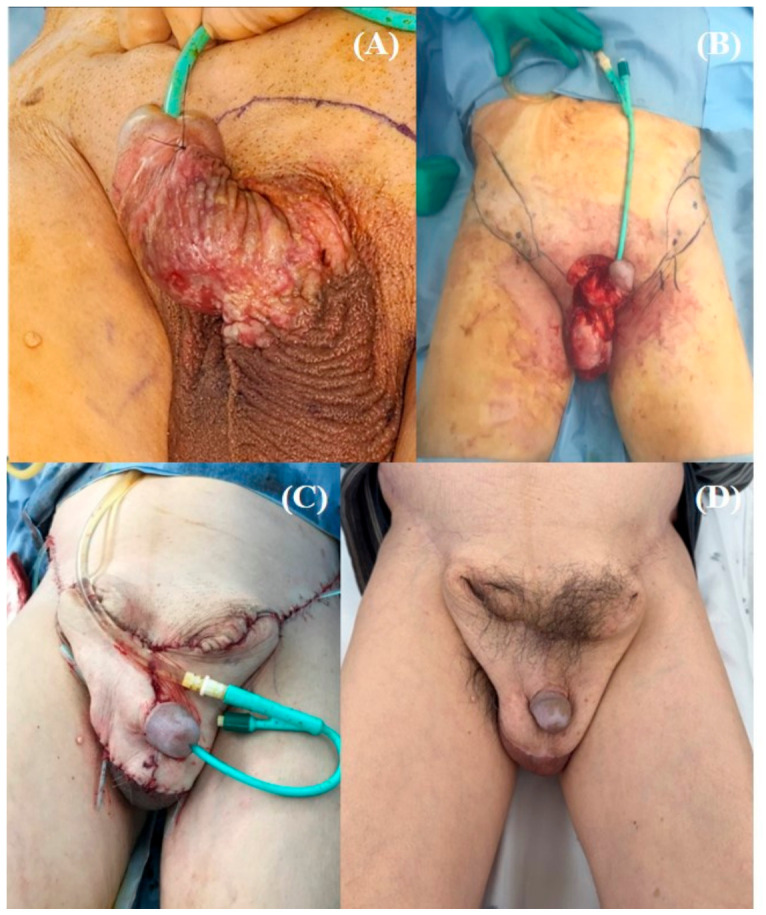
(**A**) Bilateral pedicled SCIP case of an 87-year-old male with punch-biopsy proven EMPD, widely distributed at the penis and superior scrotum. (**B**) Wide excision of penile and scrotal skin. (**C**) Immediate postoperative view. (**D**) 20-month postoperative view.

**Figure 4:**
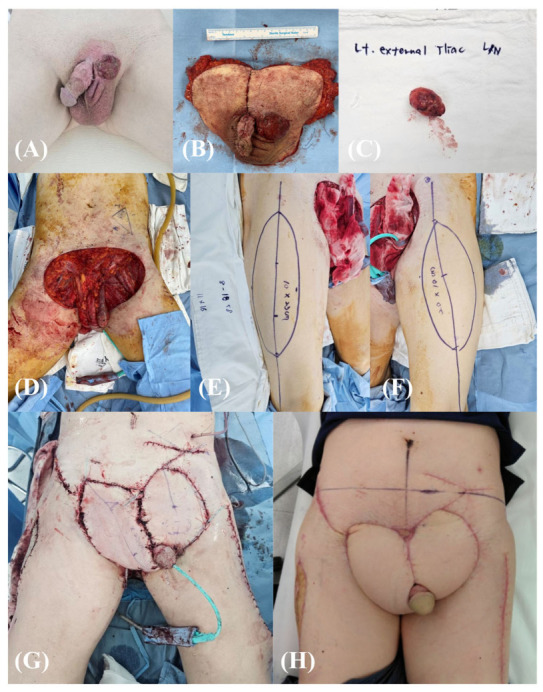
(**A**) Bilateral pedicled ALT case of a 67-year-old male with punch-biopsy proven EMPD at left groin. (**B**) Wide excision of tumor, including penile and superior scrotal skin. (**C**) Enlarged external iliac lymph node was dissected. (**D**) Large defect at the penis, scrotum, and groin after wide excision. (**E**) A pedicled ALT flap was harvested from the right anterolateral thigh. (**F**) Another pedicled ALT flap was harvested from the left anterolateral thigh. (**G**) Immediate postoperative view. (**H**) 2-month postoperative view.

**Figure 5:**
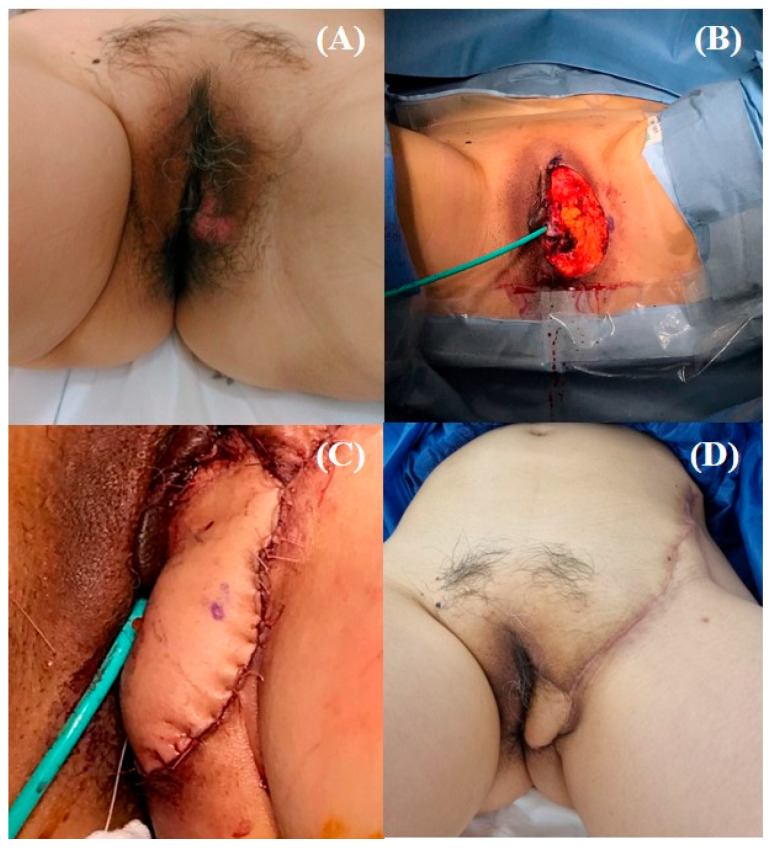
(**A**) Unilateral pedicled SCIP case of a 53-year-old female with punch-biopsy proven EMPD, widely distributed at the left perineum. (**B**) Wide excision of the left perineum. (**C**) Immediate postoperative view. (**D**) 15-month postoperative view.

**Figure 6:**
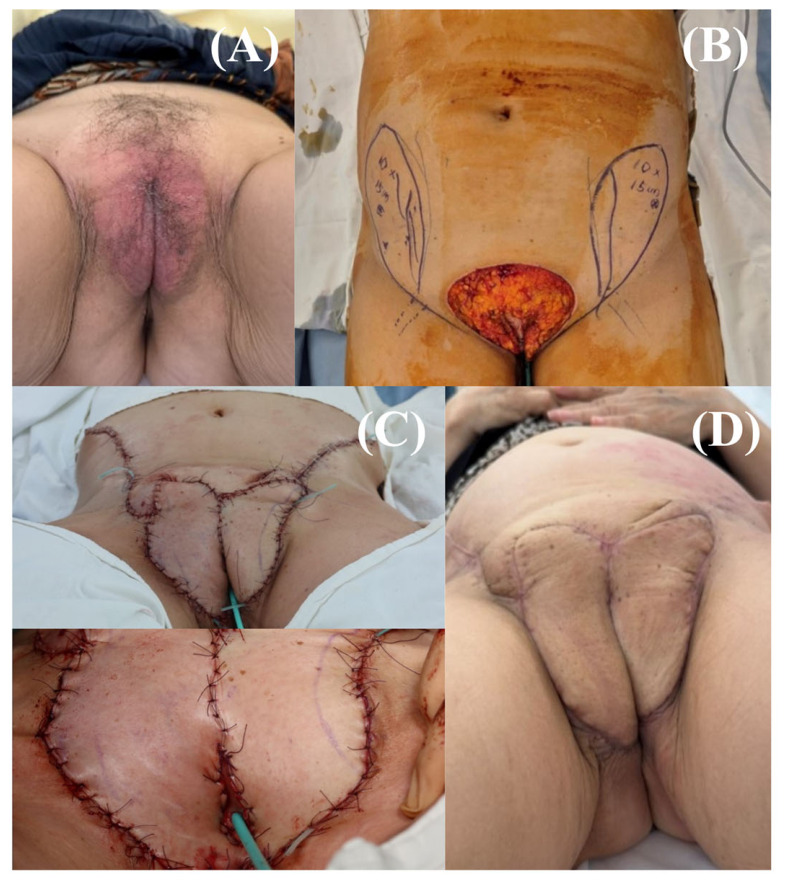
(**A**) Bilateral pedicled SCIP case of a 68-year-old female with punch-biopsy proven EMPD, widely distributed at the bilateral perineum. (**B**) Wide excision including both perinea. (**C**) Immediate postoperative view. (**D**) 3-month postoperative view.

**Figure 7:**
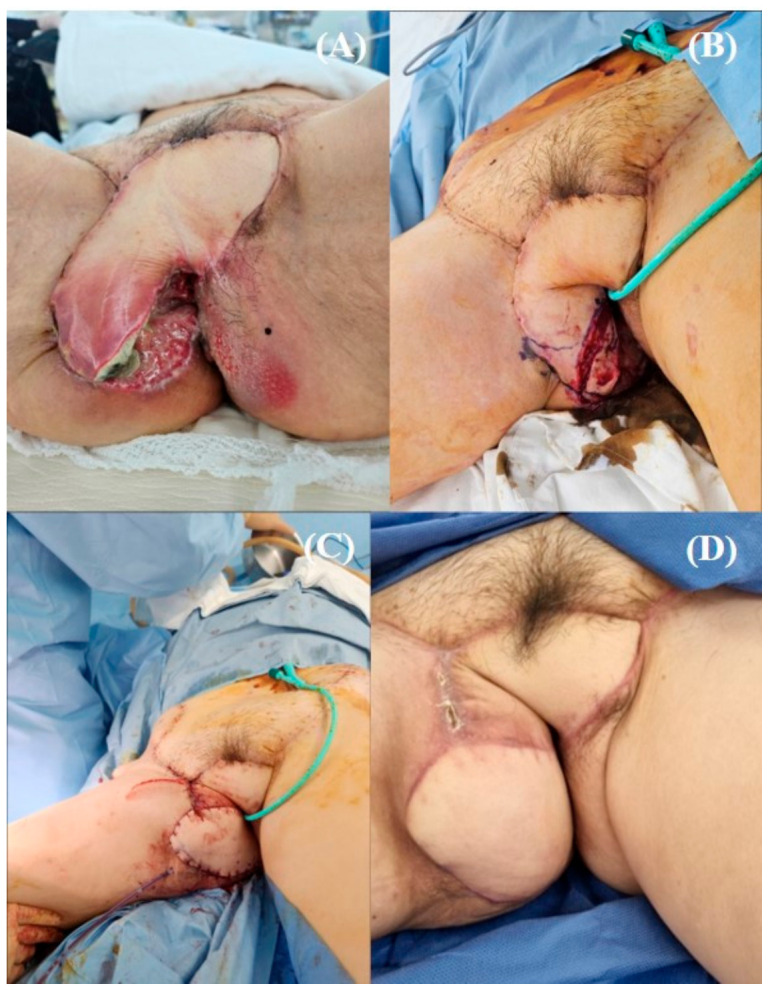
(**A**) Unilateral pedicled ALT case of a 65-year-old female who underwent previous excision by a gynecologic surgeon with a 3 o’clock margin and vaginal margin involved, 2-week postoperative view. (**B**) Design of wide excision, including further vaginal margin and previous SCIP flap. (**C**) Immediate postoperative view, using right ALT flap. (**D**) 5-month postoperative view.

Fourteen of 15 patients (93.3%) achieved negative margins on final histopathologic examination following frozen-section guided re-excision when necessary. One patient, who had preoperative pathologic lymph node involvement, had a persistently involved deep margin that was not amenable to further re-excision due to proximity to vital structures. This patient was referred for adjuvant oncologic management but subsequently developed local recurrence and distant metastasis. During a mean follow-up of 17.7 months (SD 1.3, range 7–24), one patient (6.7%) who had developed distant metastasis subsequently had a tumor-related death. Among the 14 surviving patients, 13 (92.9%) reported overall satisfaction with their postoperative appearance and functional capacity (median 4, IQR 3–4, range 1–4) (example cases, Figure 2, Figure 3, Figure 4, Figure 5, Figure 6 and Figure 7).

On outpatient examination, the reconstructed perineal region demonstrated good tissue elasticity, minimal scarring, natural contour, and preserved urination comparable to preoperative status. The thin, pliable nature of the SCIP flap closely matched native perineal tissue in texture and contour, contributing to favorable aesthetic outcomes in the SCIP group. In the ALT group, no patient reported scrotal thermal discomfort or functional limitations during activities of daily living.

## 4. Discussion

Wide local excision for localized EMPD commonly leaves extensive superficial defects of the penis, scrotum, perineum, and adjacent pubic skin, and reconstruction must restore durable coverage while preserving elasticity, contour, and thermoregulatory function in a sensitive anatomic region [34]. This descriptive case series evaluated a reconstructive selection strategy using pedicled SCIP and ALT flaps, demonstrating that tailored flap selection based on defect characteristics enabled reliable coverage and generally favorable postoperative outcomes across a spectrum of defect sizes and topography.

The selection of pedicled perforator flaps over alternative reconstructive options in this cohort was based on the need to provide thin, pliable tissue that mimics the natural contour of the perineum while avoiding the morbidity associated with more traditional approaches. Split-thickness skin grafting, while appropriate for smaller, well-approximated defects, is associated with contracture and impaired function in larger scrotal defects [35]. Myocutaneous flaps, including vertical rectus abdominis musculocutaneous (VRAM) and TFL, carry donor-site morbidity, including abdominal wall complications (e.g., abscesses, hernias, fistula, etc.) and excessive bulk that may compromise the natural contour of the donor site [11,17,23]. Free tissue transfer, while versatile, necessitates microsurgical anastomosis and prolonged operative time [26]. Pedicled perforator flaps avoid these trade-offs, offering well-vascularized tissue with minimal donor-site morbidity [16,21,26,31].

### 4.1. Technical Considerations: SCIP Versus ALT Flap Selection

Technical selection between SCIP and ALT should be based on defect size, depth, and topography. In this case series, the SCIP flap was selected for superficial to moderate depth defects involving the penile shaft, scrotum, or adjacent pubic region, where thin, pliable coverage was the primary objective [33]. However, because the SCIP flap provides a limited tissue volume, it makes it less suitable for large or deeply excavated defects. In this case series, all 10 SCIP flaps were utilized for defects of moderate size with satisfactory outcomes, consistent with its known utility for two-dimensional defects requiring contour preservation without excessive bulk [27,28].

The ALT flap was reserved for defects exceeding the tissue volume available from the SCIP or for deeper, three-dimensional losses involving the perineum or vulva that required robust volume to obliterate dead space and restore functional anatomy [18,36]. The five ALT flaps in this series had a mean area nearly 2.5 times larger than the SCIP group, reflecting the selection bias inherent in this descriptive approach. The well-characterized vascular anatomy of the descending branch of the lateral circumflex femoral artery provides a reliable pedicle, and the ALT’s versatility in terms of flap dimensions enables reconstruction of a wide range of defect geometries [19,21,30,31]. However, the potential disadvantages for using ALT flaps include donor-site morbidity on the thigh and the need for intraoperative thinning to avoid excessive bulk in genital reconstruction [8].

For extensive defects spanning multiple anatomic subregions (e.g., combined inguinal, scrotal, and perineal involvement), the operative algorithm in this series supported a staged approach: unilateral reconstruction for smaller defects and bilateral reconstruction when the defect was larger or more extensive [33]. Preoperative CTA and intraoperative Doppler mapping were used for both flaps to identify reliable perforators and plan the pedicle. These technical adjuncts are particularly important in patients with prior surgical scarring or altered vascular anatomy.

### 4.2. Oncologic Safety

Short-term oncologic safety was observed in the majority of this cohort. While 14 of 15 patients achieved negative margins, the 18-month mean follow-up is insufficient to conclude durable safety given the known propensity for late recurrence in EMPD. The observed short-term local recurrence rate of 6.7% compares favorably with previously reported rates for perineal EMPD, which range from 8% to 61% depending on margin status and follow-up duration [5,6,37]. Importantly, reconstruction did not appear to compromise the ability to obtain margin-negative resection, and the low rate of major flap-related complications suggests that immediate reconstruction can be performed safely after adequate oncologic clearance.

### 4.3. Limitations and Future Direction

The primary limitation of this study is its small sample size (*n* = 15) and short mean follow-up (18 months), which are insufficient to claim durable oncologic safety. Additionally, patient satisfaction and functional outcomes were assessed using a simple, non-validated ordinal scale by the attending surgeon, and the non-randomized assignment of flap types according to defect characteristics precludes direct statistical comparison between groups. As such, future studies should validate and further refine the selection algorithm in larger multicenter cohorts and compare SCIP and ALT directly with skin grafting, VRAM, TFL, and IPAP reconstruction using standardized patient-reported cosmetic and functional outcomes, along with longer oncologic surveillance.

## 5. Conclusions

This descriptive case series suggests that pedicled SCIP and ALT flap reconstruction after wide excision of perineal EMPD provides reliable, well-vascularized tissue coverage with high flap survival, low complication rates, and generally favorable cosmetic and functional outcomes based on short-term follow-up. Pedicled SCIP flaps are ideally suited for defects requiring thin, pliable coverage with minimal donor-site morbidity, while pedicled ALT flaps offer greater tissue volume for larger or more complex defects. When necessary, combination with bilateral pedicled scrotal flaps and selective skin grafting allows tailored reconstruction of extensive, three-dimensional defects while maintaining oncologic safety through frozen-section-guided margin control. A flap selection strategy based on defect size, location, and tissue requirements enables optimized outcomes across the full spectrum of perineal EMPD defects.

## Figures and Tables

**Table 1:** Patient and lesion characteristics.

Outcomes	Value *N* = 15
**Age (years)**	63 (7.3, 51–78) ^1^
**Sex**	
Female	4 (26.7%)
Male	11 (73.3%)
**Comorbidities ^†^**	
None	7 (46.7%)
Hypertension	4 (26.7%)
Diabetes Mellitus	3 (20%)
Dyslipidemia	1 (6.7%)
Chronic Kidney Disease	1 (6.7%)
Previous EMPD Excision	1 (6.7%)
**Lesion Location ^†^**	
Penile Region	6 (40%)
Scrotal Region	7 (46.7%)
Perineal Region	10 (66.7%)
Pubic/Groin Region	5 (33.3%)
Vaginal Region	1 (6.7%)
**Lesion Distribution**	
Bilateral	6 (40%)
Unilateral	9 (60%)
**Preoperative Lymph Node Status**	
Enlarged	1 (6.7%)
Not Enlarged	14 (93.3%)
**Pathologic Lymph Node Involvement**	
Yes (metastatic)	1 (6.7%)
N/A	14 (93.3%)
**Symptom Duration Prior Diagnosis (months)**	14 (9–24, 5–84) ^2^

^1^ Mean (SD, range); ^2^ Median (IQR, range). ^†^ The sum of percentages exceeds 100% due to multiple responses per patient.

**Table 2:** Perioperative characteristics.

Outcomes	Value *N* = 15
**Surgical Position**	
Lithotomy	3 (20%)
Supine	12 (80%)
**Excision Margin (cm)**	2 (2–2, 2–3) ^2^
**Operative Time (mins)**	180 (155–265, 140–310) ^2^
**Flap Type**	
Pedicled SCIP Flap	10 (66.7%)
Pedicled ALT Flap	5 (33.3%)
**Flap Laterality**	
Unilateral	9 (60%)
Bilateral	6 (40%)
**Harvest Side**	
Left	3 (20%)
Right	6 (40%)
Both	6 (40%)
**Flap Area (cm^2^)**	
SCIP Flap	106 (23.3, 30–240) ^1^
ALT Flap	245.2 (41.2, 120–338) ^1^
**Preoperative Lymph Node Status**	
Enlarged	1 (6.7%)
Not Enlarged	14 (93.3%)
**Donor Site Closure**	
Primary Closure	14 (93.3%)
Additional STSG	1 (6.7%)

^1^ Mean (SD, range); ^2^ Median (IQR, range).

**Table 3:** Postoperative outcomes.

Outcomes	Value *N* = 15
**Postoperative Complications**	
Total Flap Loss	0
Partial Flap Necrosis	1 (6.7%)
Donor-site: Primary Closure	14 (93.3%)
Donor-site: STSG Required	1 (6.7%)
DVT/PE	0
Major Complications (contracture, voiding, erectile dysfunction)	0
**Oncological Outcomes**	
Negative Margins Achieved	14 (93.3%)
Local Recurrence/Distant Metastasis	1 (6.7%)
Tumor-related Death	1 (6.7%)
**Follow-Up Duration (months)**	17.7 (1.3, 7–24) ^1^
**Patient-Reported Satisfaction**	4 (3–4; 1–4) ^2^

^1^ Mean (SD, range); ^2^ Median (IQR, range).

## Data Availability

The data presented in this study are available on request from the corresponding author due to patient confidentiality and privacy restrictions.

## Supplementary Materials

The following supporting information can be downloaded at: https://www.mdpi.com/article/10.3390/medicina62071291/s1, Table S1: Demographic and surgical variables of patients with penoscrotal EMPD.

## Abbreviations

The following abbreviations are used in this manuscript:

EMPD	Extramammary Paget’s disease
ALT	Anterolateral thigh
TFL	Tensor fascia lata
SCIP	Superficial circumflex iliac artery perforator
SCIA	Superficial circumflex iliac artery
VRAM	Vertical rectus abdominis musculocutaneous
IPAP	Internal pudendal artery perforator
