# Supplementary material for: Wide Excision and Flap Reconstruction in Perineal Extramammary Paget’s Disease Patients
Source: Medicina (Kaunas). 2026 Jul 3;62(7):1291. doi: 10.3390/medicina62071291 (PMC13414416; doi:10.3390/medicina62071291)
Supplement: Supplementary file 1 [file medicina-62-01291-s001.zip › medicina-4343057-supplementary.pdf]

Table S1. Demographic and surgical variables of patients with penoscrotal EMPD.

Patient	Age (years)	Comorbidities	Defect location	Defect size (cm)	Reconstructive method	Flap size (cm)	Complication	Follow-up (months)
1	68	HTN	Scrotum	8 × 10	Pedicled SCIP	17 × 8	None	22
2	72	HTN, CHD	Penoscrotal and suprapubic	8 × 18	Pedicled SCIP + skin graft	20 × 7 + 18 × 5	None	24
3	64	None	Penoscrotal, perineum and hypogastric	12 × 15	Pedicled SCIP + skin graft	21 × 7 + 21 × 7	Skin graft partial loss	20
4	59	DM	Penoscrotal	7 × 9	Pedicled SCIP	15 × 7	None	18
5	66	HTN	Scrotum and perineum	9 × 11	Pedicled SCIP + skin graft	18 × 8	None	21
6	71	HTN, DM	Penoscrotal and pubic	10 × 13	Pedicled SCIP + skin graft	19 × 8 + 14 × 6	Wound dehiscence	19
7	61	None	Scrotum	5 × 6	Pedicled SCIP	10 × 5	None	24
8	57	None	Penoscrotal and pubic	9 × 12	Pedicled SCIP + skin graft	20 × 7	None	15
9	74	HTN, CHD	Penoscrotal	7 × 8	Pedicled SCIP	14 × 6	None	14
10	62	DM	Penoscrotal and perineum	10 × 14	Pedicled SCIP + skin graft	22 × 8	Distal partial necrosis; secondary ALT flap	18
11	63	HTN	Penoscrotal, perineum and pubic	14 × 16	Pedicled ALT + skin graft	26 × 9	None	20
12	52	None	Penoscrotal, perineum and hypogastric	16 × 18	Pedicled ALT + skin graft	28 × 9	Wound infection	13
13	78	HTN, DM	Penoscrotal and inguinal	11 × 13	Pedicled ALT + inguinal LN dissection + skin graft	24 × 8	None	16
14	70	CHD	Penoscrotal, pubic and inguinal	12 × 14	Pedicled ALT + inguinal LN dissection	22 × 9	Local recurrence (re-excision at 20 mo)	22
15	67	HTN	Penoscrotal and pubic	13 × 15	Pedicled ALT (secondary after SCIP partial loss)	25 × 8	None	12

CHD, coronary heart disease; DM, diabetes mellitus; EMPD, extramammary Paget's disease; HTN, hypertension; RA, rheumatoid arthritis; SCIP, superficial circumflex iliac artery perforator propeller flap.
